# Global scientific trends in laparoscopy and gastric cancer in the 21st century: A bibliometric and visual mapping analysis

**DOI:** 10.3389/fonc.2023.1136834

**Published:** 2023-02-23

**Authors:** Hongmin Han, Zhanwei Wang, Xiaodan Zhao, Guosheng Li, Yuan Fu, Zhongqing Wang, Hongyan Wang

**Affiliations:** ^1^ Department of General Surgery, The People’s Hospital of China Medical University, Shenyang, China; ^2^ Department of General Surgery, Shenzhen Hyzen Hospital, Shenzhen, China; ^3^ Department of Information Center, The First Hospital of China Medical University, Shenyang, China

**Keywords:** gastric cancer, laparoscopy, bibliometric, hotspot, trend

## Abstract

**Aims:**

To use visual mapping and bibliometrics to analyze and summarize the valuable information on laparoscopic surgery for gastric cancer (GC) obtained in the last 20 years, so as to determine the research hotspots and trends in this field.

**Methods:**

We screened all literature on laparoscopic surgery for GC in the Web of Science published from 2000 to 2022 and analyzed the research hotspots and trends in this field using VOSviewer.

**Results:**

A total of 2796 reports from 61 countries and regions were selected. Japanese researchers published the most papers (n=946), followed by those from China (n=747) and South Korea (n=557). Papers from Japan also had the most citations (n=21,836). *Surgical Endoscopy and Other Interventional Techniques* published the most reports on laparoscopic surgery for GC (n=386) and also had the highest total number of citations (n=11,076), making this journal the most authoritative in this field. Among the institutions, researchers from Seoul National University in South Korea had the highest numbers of published papers and citations. The keywords of the articles could be divided into five categories: surgical methods for GC, short-term and long-term efficacy of laparoscopic surgery, guiding role of laparoscopy in the treatment of advanced GC, diagnosis and treatment of early gastric cancer (EGC), and lymph node dissection. Keywords such as “laparoscopic proximal gastrectomy”, “surgical outcomes”, and “esophagogastric junction” have emerged recently, and relevant studies on laparoscopic surgery for adenocarcinoma of esophagogastric junction(AEG)have gradually become a hot topic and trend.

**Conclusion:**

This study adopted bibliometric analysis to identify the current research hotspots and research trends in the field of laparoscopic surgery for GC. Five main research hotspots of laparoscopic surgery for GC were also identified. Laparoscopic surgery for AEG may become an important research focus in the future.

## 1 Introduction

Gastric cancer (GC) is one of the most common malignancies globally and the fourth leading cause of cancer-related death (1). It has a very poor overall survival rate, with more than 1 million new cases and about 783,000 deaths in 2018. Most new cases emerge in Asia and South America (2, 3).

Surgery is still the most important treatment for GC. Since Kitano first reported laparoscopic-assisted gastrectomy (LAG) for early distal gastric cancer in 1994 (4), laparoscopic surgery has played an important role in treating an increasing number of cases of early and locally advanced GC. Compared with traditional laparotomy, LAG has the advantages of less bleeding, less postoperative pain, faster recovery of gastrointestinal function, and a shorter hospital stay, and its safety was also confirmed (5, 6). With the continuous improvement of medical techniques and the intensification of research in large centers, the role of laparoscopic surgery in the diagnosis and treatment of GC has diversified, and it plays different roles in the treatment strategies of different stages of GC.

In recent years, bibliometric analysis has become increasingly popular among researchers. It uses mathematical and statistical methods to carry out qualitative and quantitative analyses on the publications in databases, revealing the historical development, research focus, and future trends of a certain field. It can also systematically output valuable and reliable information from all relevant literature in a certain field in the form of visual maps and tables. With the widespread application of laparoscopic surgery for treating GC, the number of papers related to this approach has increased. However, to the best of our knowledge, no bibliometric study on laparoscopic surgery for GC has been performed, and the research hotspots and developing trends in this field have remained unclear. In this study, we reveal the trends of publications on laparoscopic surgery for GC in the last 20 years; identify influential journals, countries, institutions, and authors; explore the networks of international collaboration; and reveal research hotspots and emerging topics.

## 2 Method

### 2.1 Database selection

**Figure 1:**
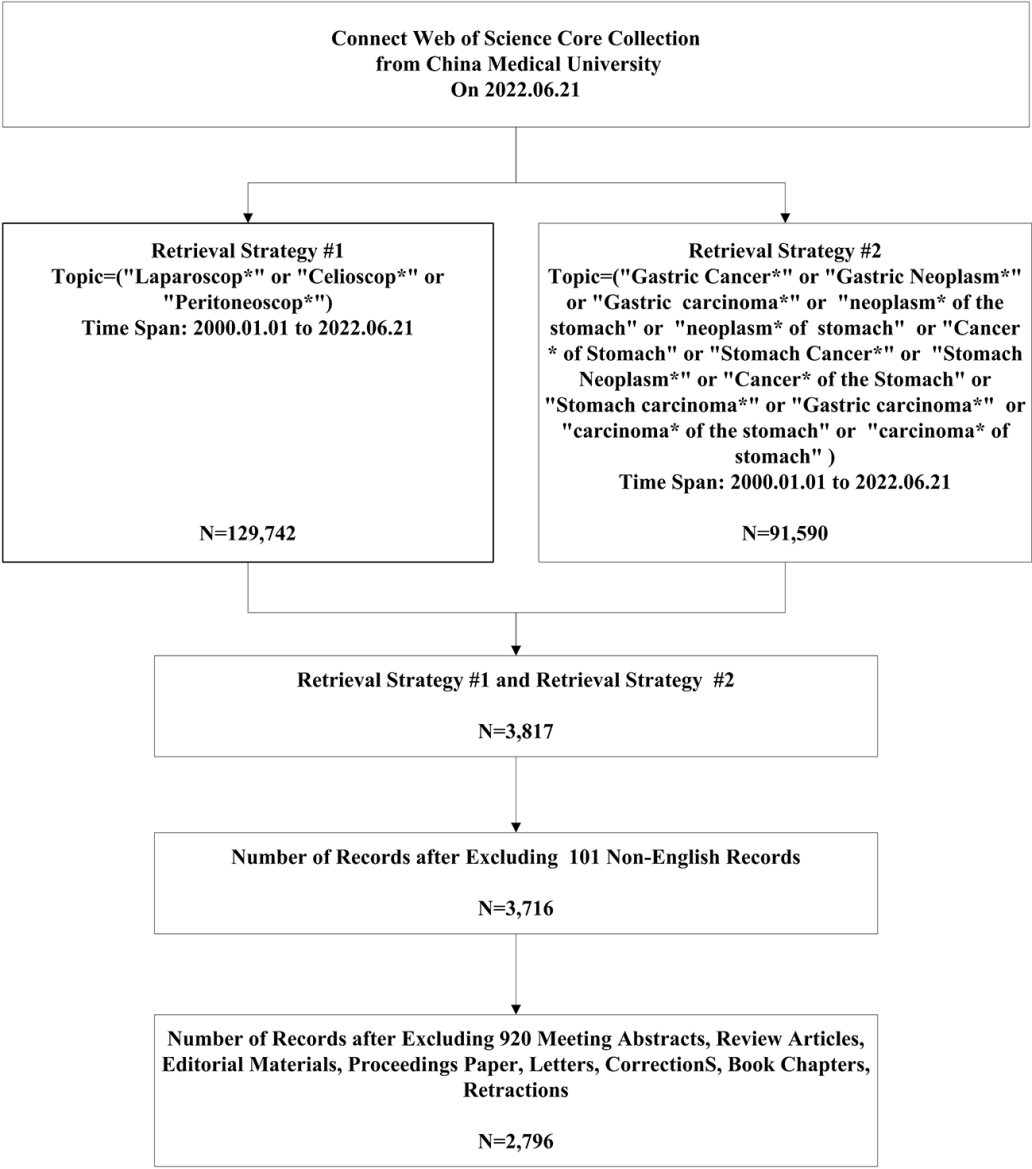
Flow chart of the search strategy. "*" can represent any length of character group.

Web of Science is the most comprehensive academic database covering the most subjects globally, and has been widely used in bibliometric analysis. To avoid any impact of database updates on this study, all bibliometric data were here downloaded by year on June 21, 2022. The search criteria were as follows: The literature publication period was from January 1, 2000, to June 21, 2022; the type of articles to be searched was limited to Article; and the language of the articles was limited to English. The following search terms were used: TS=(“Gastric Cancer*” or “Gastric Neoplasm*” or “Gastric carcinoma*” or “neoplasm* of the stomach” or “neoplasm* of stomach” or “Cancer* of Stomach” or “Stomach Cancer*” or “Stomach Neoplasm*” or “Cancer* of the Stomach” or “Stomach Carcinoma*” or “Gastric Carcinoma*” or “Carcinoma* of the Stomach” or “Carcinoma* of Stomach”) and TS=(“Laparoscop*” or “Celioscop*” or “Peritoneoscop*”). The screening process is shown in **Figure 1**.

### 2.2 Analytical tools and methods

VOSviewer is classical bibliometric analysis software that is widely used in literature analysis research. It can support multiple types of bibliometric studies, including co-authorship, co-citation, and co-occurrence analyses. This study used VOSviewer 1.6.18 for the analyses. First, co-authorship analysis of the organization and author was performed, and a visual map was established. Then, co-occurrence analysis of high-frequency keywords was carried out, and a visual map of such keywords was established. Descriptive analyses of the year of publication, journal, country, institution, authors, and references were also performed.

## 3 Result

### 3.1 Publication volume by year

**Figure 2:**
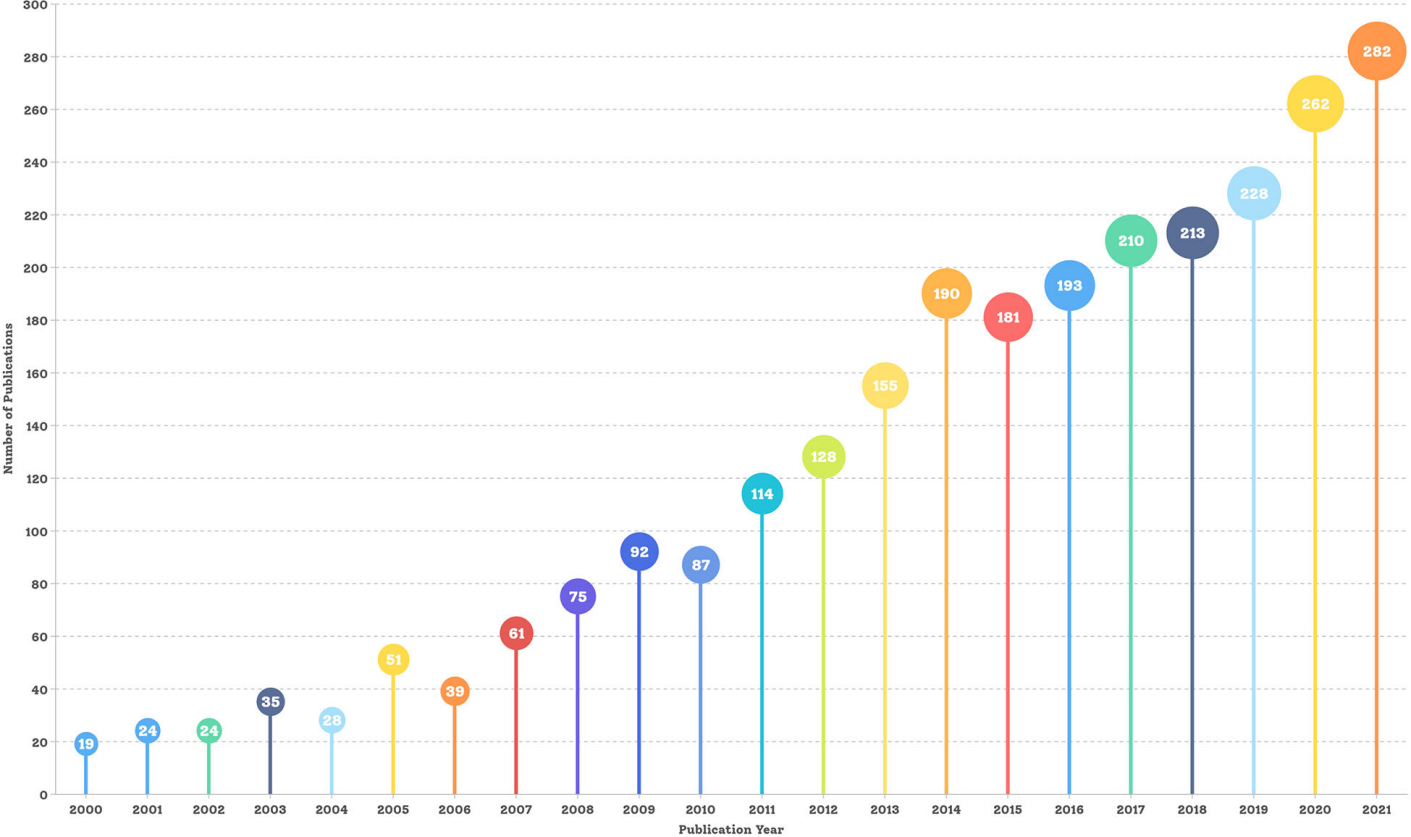
Annual number of publications and growth trends from 2000 to 2021.

According to the inclusion criteria, a total of 2796 articles related to laparoscopic surgery for GC were retrieved and included in the final analysis. The trend in the number of publications for 2000–2021 is shown in **Figure 2** (the data for 2022 are not complete, so this year is not included in this figure), indicating a general increase in the number of articles. In 2000, only 19 research publications related to laparoscopic surgery for GC were published, while in 2011 more than 100 were published within a year for the first time. The number of papers peaked in 2021 at 282, which was 14.48 times the level in 2000. This indicates that laparoscopic surgery for GC is receiving increasing attention.

### 3.2 Journal of publication

A total of 321 journals published articles on laparoscopic surgery for GC between 2000 and 2022. **Table 1** lists the top 10 journals with the highest number of publications and related impact indicators. The 10 most active journals in this field have published 1202 related articles, accounting for 42.99% of the total. *Surgical Endoscopy and Other Interventional Techniques* was the most productive journal in this field with 386 papers, along with a total of 11,076 citations, making it also the most cited journal in this field. The editors of this journal clearly have a great interest in this field and are willing to receive articles related to it; the quality of such articles is also high. *Journal of Gastric Cancer* has the most recent average publication year, indicating that the number of articles related to laparoscopic surgery for GC published in this journal has increased rapidly in recent years.

**Table 1:** The top 10 journals in the field of laparoscopic surgery for GC.

Rank	Journal	IF	Counts	Citations	Avg.Citations	Avg.Pub.Year
1	*Surgical Endoscopy And Other Interventional Techniques*	3.453	386	11076	29	2014.17
2	*Gastric Cancer*	7.701	125	3605	29	2015.34
3	*Surgical Laparoscopy Endoscopy and Percutaneous Techniques*	1.455	117	1319	11	2013.13
4	*Annals Of Surgical Oncology*	4.339	113	3542	31	2015.00
5	*World Journal Of Gastroenterology*	5.371	97	2118	22	2014.67
6	*Journal Of Gastric Cancer*	3.197	78	601	8	2018.04
7	*Journal Of Laparoendoscopic and Advanced Surgical Techniques*	1.766	77	853	11	2014.99
8	*Journal Of Gastrointestinal Surgery*	3.267	76	1542	20	2015.25
9	*World Journal Of Surgery*	3.282	69	2203	32	2013.28
10	*Hepato-Gastroenterology*	0.8	64	548	9	2010.20

IF,impact factor

### 3.3 Countries of publication

Overall, researchers from 61 countries and regions have participated in studies of laparoscopic surgery for GC. **Table 2** lists the top 10 countries in terms of publication volume. Researchers in Japan have been the most productive (n=946 publications), followed by those in China (n=747) and South Korea (n=557). Japan is also the country with the most citations (n=21,836). Among the top 10 countries, the Netherlands and China have the most recent average publication years.

**Table 2:** The top 10 countries with the most publications in the field of laparoscopic surgery for GC.

Rank	Country	Counts	Citations	Avg.Citations	Avg.Pub.Year
1	JAPAN	946	21836	23	2014.03
2	CHINA	747	8467	11	2017.51
3	SOUTH KOREA	557	14767	27	2014.94
4	USA	196	4681	24	2013.53
5	ITALY	97	2788	29	2014.27
6	ENGLAND	58	1461	25	2012.55
7	GERMANY	51	1264	25	2011.25
8	NETHERLANDS	34	642	19	2017.61
9	TURKEY	32	366	11	2016.57
10	FRANCE	28	839	30	2013.82

**Figure 3:**
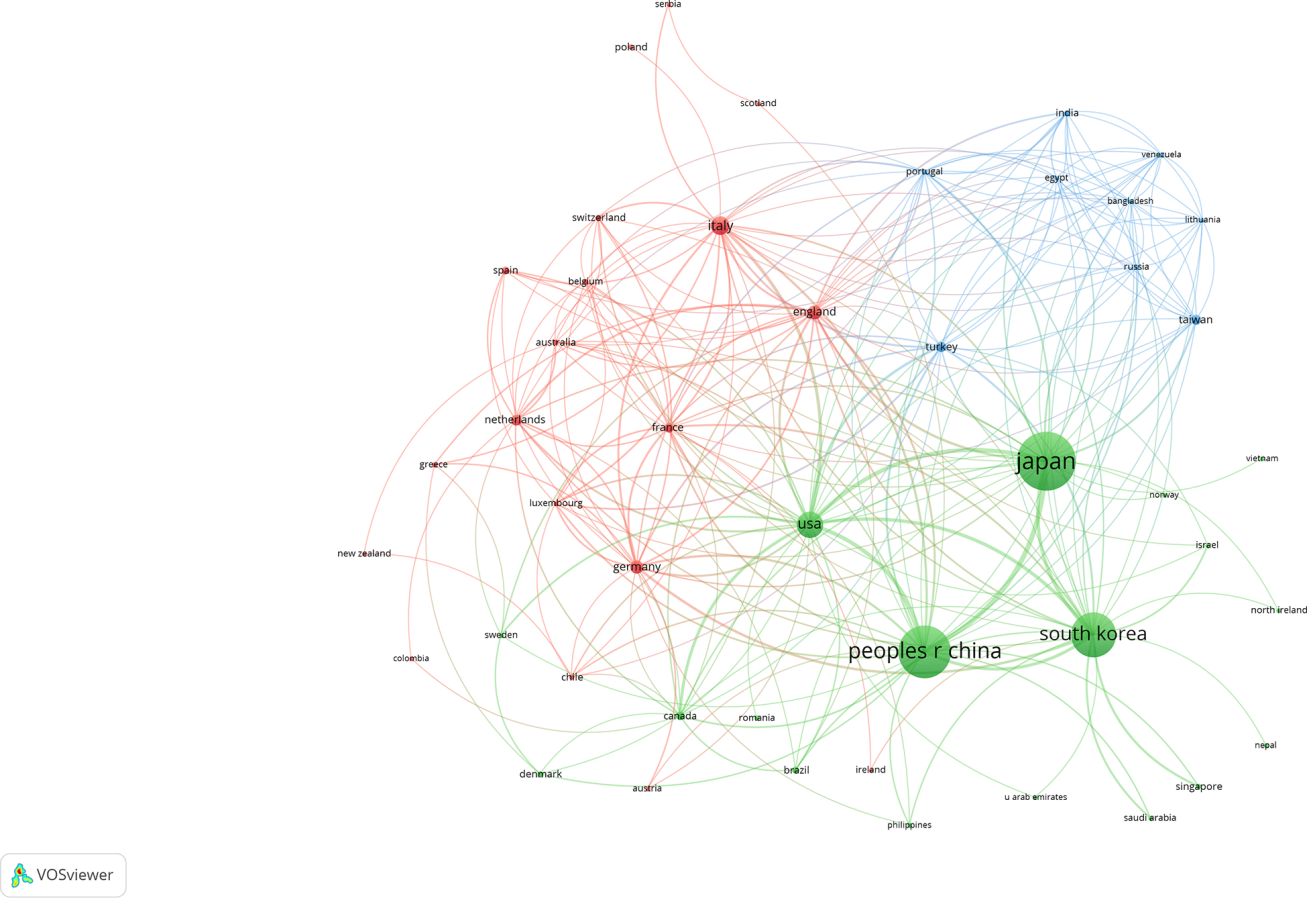
Collaboration between countries based on VOSviewer.

We used VOSviewer to perform co-authorship analysis in 61 countries. As shown in **Figure 3**, researchers in 46 countries form the largest co-authorship network, which consists of three clusters. Researchers in the United States work with others in 32 countries and regions, followed by those in South Korea (n=30), and China and Italy (both n=27). The results show that researchers in the United States are particularly active in international collaboration.

### 3.4 Institution of publication

According to our statistics, researchers at a total of 1756 institutions participated in the publication of papers on laparoscopic surgery for GC. The top 10 institutions are shown in **Table 3**. Seoul National University was the institution with the most associated publications (n=135), followed by Yonsei University (n=103) and Fujian Medical University (n=99). Seoul National University was also the institution associated with the most citations (n=5262), followed by Yonsei University (n=4931) and National Cancer Centre Korea (n=3507). The average citations of Ajou University was the highest, at 51. Meanwhile, the average publication year of Fujian Medical University was the most recent (2017.61). These results show that Seoul National University is the most authoritative institution in this field. Meanwhile, Ajou University’s research results are of particularly high quality, while Fujian Medical University has good research potential in this field.

**Table 3:** The top 10 institutions in terms of articles published in the field of laparoscopic surgery for GC.

Rank	Institution	Counts	Citations	Avg.Citations	Avg.Pub.Year
1	Seoul National University	135	5262	39	2015.03
2	Yonsei University	103	4931	48	2014.63
3	Fujian Medical University	99	1719	17	2017.61
4	The Catholic University of Korea	98	3111	32	2015.31
5	National Cancer Centre Korea	80	3507	44	2015.08
6	Ajou University	63	3208	51	2015.90
7	Japanese Foundation For Cancer Research	57	1399	25	2015.70
8	Zhejiang University	51	532	10	2016.63
9	Chonnam National University	51	2118	42	2015.82
10	University of Ulsan	47	764	16	2015.62

**Figure 4:**
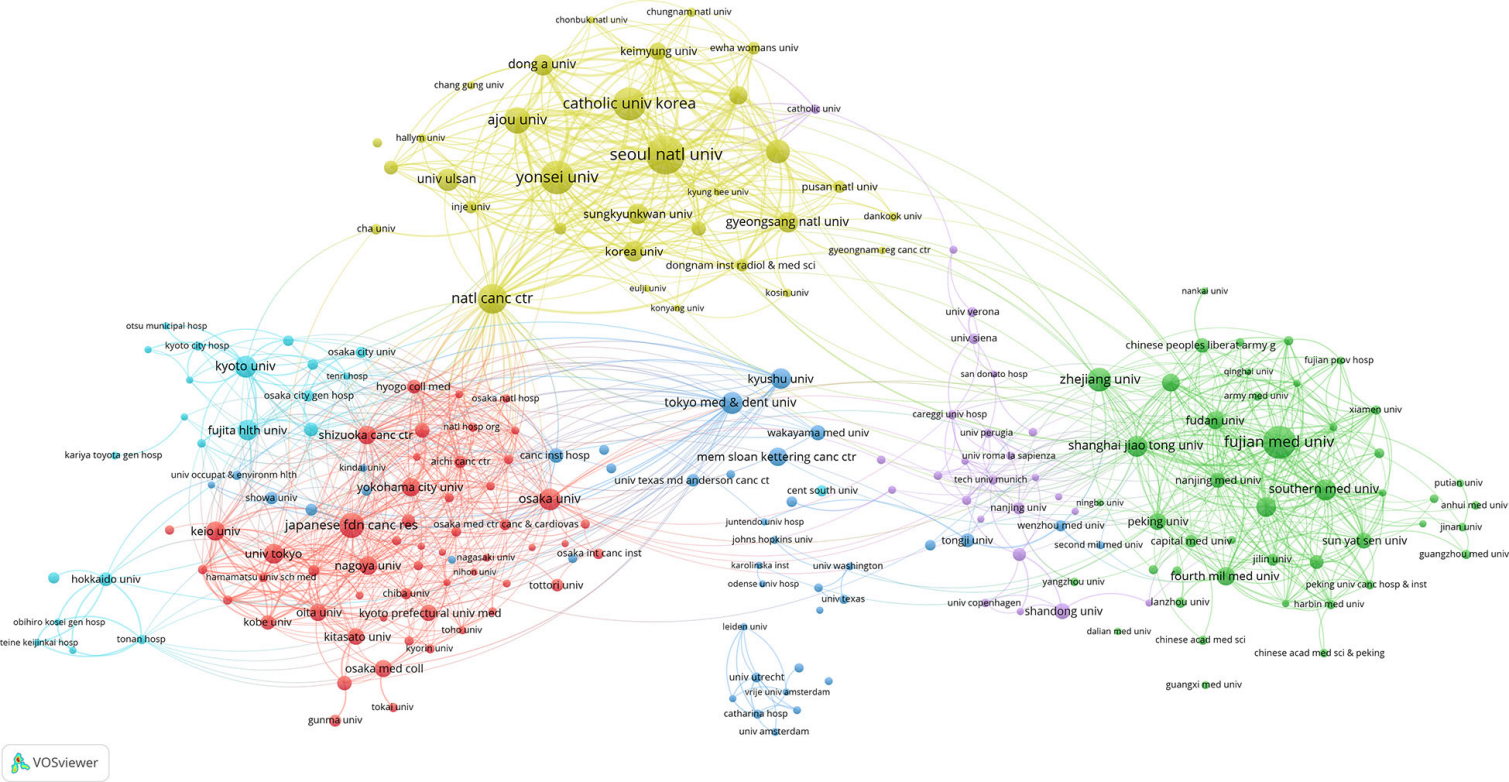
Collaboration between the institutions based on VOSviewer.

Upon setting the threshold for institutional publications at 5, we identified 232 high-yield institutions from among 1756 institutions, and performed a co-authorship analysis of these 232 institutions using VOSviewer. As shown in **Figure 4**, 217 out of the 232 high-yield institutions formed the largest institutional co-authorship network, consisting of six clusters. The National Cancer Centre Korea was suggested to be the most influential institution in this context, which collaborated with 59 high-yield institutions. This was followed by Shanghai Jiao Tong University and Shizuoka Cancer Center, each of which collaborated with 46 high-yield institutions.

### 3.5 Analysis of authors

**Table 4** shows the top 10 authors among 10,641 authors involved in publishing papers on laparoscopic surgery for GC.Hyung-Ho Kim was the author with the most publications (n=76), followed by Woo-Jin Hyung (n=74), Huang, Chang-Ming and Zheng, Chao-Hui (both n=71). Notably, two authors were cited more than 3000 times, namely, Woo-Jin Hyung (n=4166) and Hyung-Ho Kim (n=3760), indicating that their research results are widely recognized and they may be particularly prominent within the research field of laparoscopic surgery for GC.

**Table 4:** The top 10 authors in the research field of laparoscopic surgery for GC.

Rank	Author	Counts	Citations	Avg.Citations	Avg.Pub.Year
1	Hyung-Ho Kim	76	3760	49	2014.47
2	Woo-Jin Hyung	74	4166	56	2015.28
3	Huang, Chang-Ming	71	900	13	2017.20
4	Zheng, Chao-Hui	71	900	13	2017.20
5	Lin, Jian-Xian	69	896	13	2017.09
6	Xie, Jian-Wei	67	891	13	2017.01
7	Li, Ping	67	918	13	2017.04
8	Wang, Jia-Bin	66	855	13	2017.06
9	Do-Joong Park	65	2306	35	2015.42
10	Lu, Jun	64	799	12	2017.22

**Figure 5:**
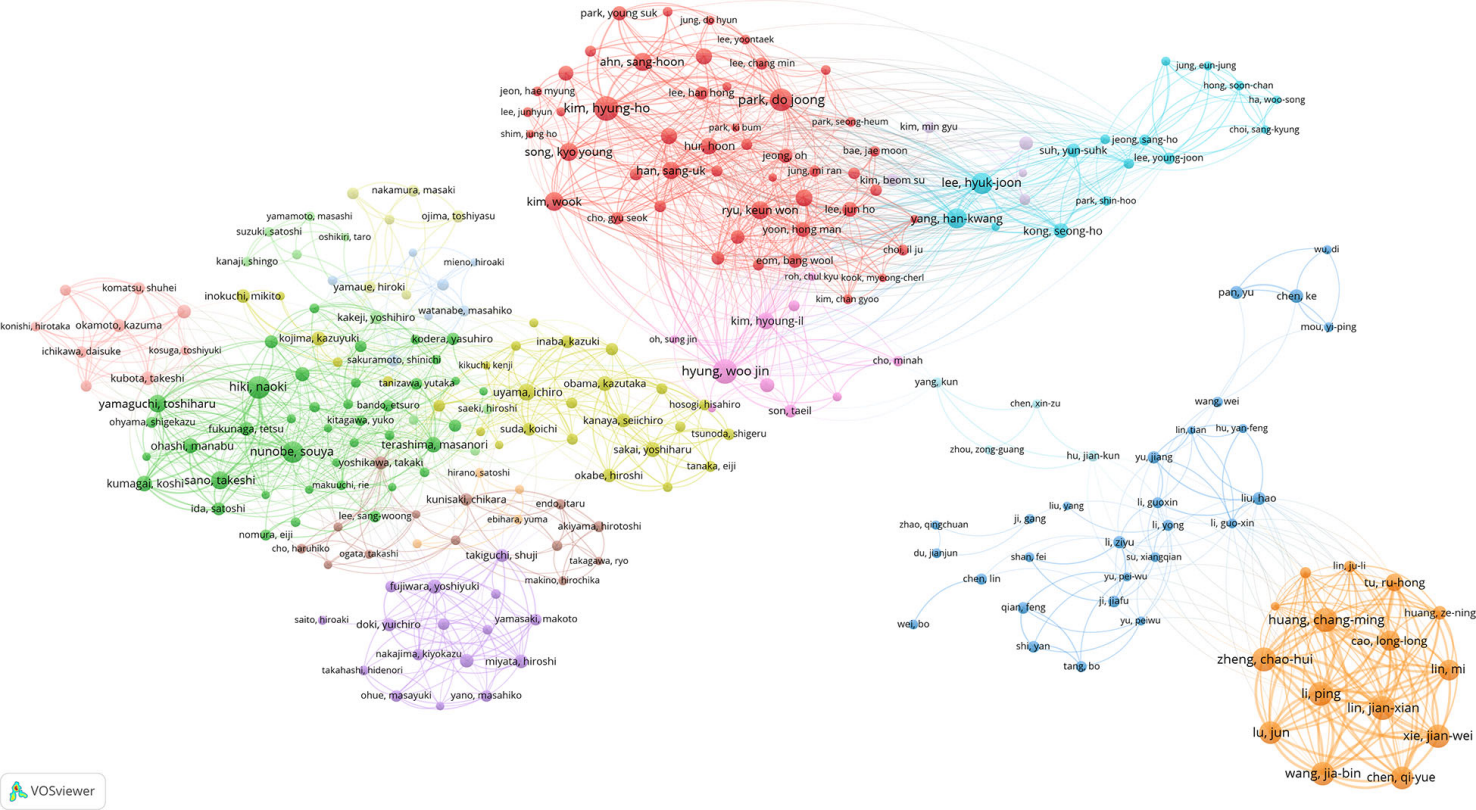
Collaboration between authors based on VOSviewer.

We set the threshold of author publications to 10, identified 258 high-yield authors from 10,641 authors, and performed co-authorship analysis on these 258 authors using VOSviewer. As shown in **Figure 5**, 255 of the 258 high-yield authors formed the largest co-author network, consisting of 16 clusters. Woo-Jin Hyung collaborated with 47 high-yield authors, followed by Hiki Naoki with 44 and Terashima Masanori with 39.

### 3.6 Highly cited literature

We list the top 10 publications in terms of total citations in **Table 5**. The total number of citations of these 10 articles was 3797, and 80% of the articles were published in *Annals of Surgery* or *Gastric Cancer*. Overall, 6 of these 10 articles were from Japan, 2 from South Korea, and the remaining 2 from Switzerland and Italy. The predominance of Asian research institutions in these statistics is reasonable given the extremely high incidence of GC in Asia.

**Table 5:** The top 10 most cited references in the field of laparoscopic surgery for GC.

Rank	PY	First Author	Title	Journal	TC	Countries/regions
1	1994	Kitano S	Laparoscopy-assisted Billroth I gastrectomy	Surg Laparosc Endosc	616	Japan
2	2004	Dindo D	Classification of surgical complications: a new proposal with evaluation in a cohort of 6336 patients and results of a survey	Annals of Surgery	485	Switzerland
3	2011	Japanese Gastric Cancer Association	Japanese classification of gastric carcinoma: 3rd English edition	Gastric Cancer	392	Japan
4	2011	Japanese Gastric Cancer Association	Japanese gastric cancer treatment guidelines 2010 (ver.3)	Gastric Cancer	388	Japan
5	2005	Huscher CG	Laparoscopic versus open subtotal gastrectomy for distal gastric cancer: five-year results of a randomized prospective trial	Annals of Surgery	371	Italy
6	2010	Kim HH	Morbidity and mortality of laparoscopic gastrectomy versus open gastrectomy for gastric cancer: an interim report–a phase III multicenter, prospective, randomized Trial (KLASS Trial)	Annals of Surgery	339	Korea
7	2007	Kitano S	A multicenter study on oncologic outcome of laparoscopic gastrectomy for early cancer in Japan	Annals of Surgery	328	Japan
8	2017	Japanese Gastric Cancer Association	Japanese gastric cancer treatment guidelines 2014 (ver.4)	Gastric Cancer	327	Japan
9	2002	Kitano S	A randomized controlled trial comparing open vs. laparoscopy-assisted distal gastrectomy for the treatment of early gastric cancer: an interim report	Surgery	284	Japan
10	2008	Kim YW	Improved quality of life outcomes after laparoscopy-assisted distal gastrectomy for early gastric cancer: results of a prospective randomized clinical trial	Annals of Surgery	267	Korea

Notably, Kitano S. wrote 3 of the 6 Japanese articles, and the article with the highest number of citations was also written by Kitano S. in 1994. He first proposed laparoscopic-assisted distal gastrectomy(LADG) for early gastric cancer(EGC). These findings highlight his major contribution to and influence in this field.

### 3.7 Keyword analysis

**Figure 6:**
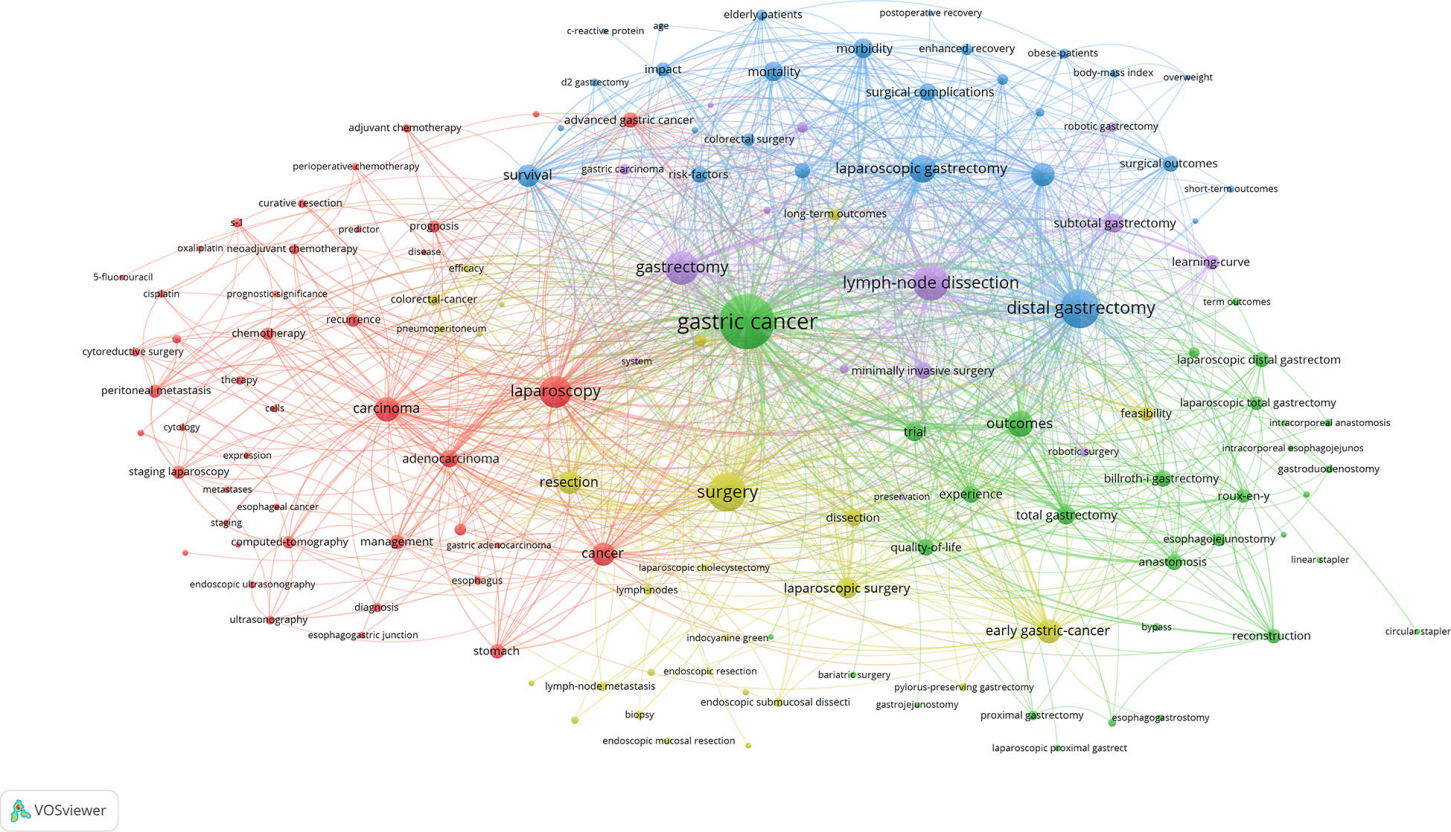
Keyword cluster analysis and co-occurrence analysis based on VOSviewer.

We extracted a total of 4882 keywords from the 2796 articles. To more intuitively and quickly obtain the trends and hotspots of laparoscopic surgery for GC, we set the keyword threshold to 20, conducted co-occurrence analysis on the 143 high-frequency keywords that met this threshold, and constructed a co-occurrence network diagram of high-frequency keywords. As shown in **Figure 6**, these high-frequency keywords are mainly divided into five clusters, represented by different colors. The red cluster represents the guiding effect of laparoscopy in the treatment of advanced GC, such as staging laparoscopy and adjuvant treatment. The green cluster represents surgical methods for GC, such as Billroth I gastrectomy and anastomotic reconstruction. The blue cluster represents the short-term and long-term efficacy of laparoscopic surgery, such as complications and mortality. The yellow cluster represents treatment of EGC, such as ESD and lymph node metastasis. Finally, the purple cluster represents lymph node dissection, such as D2 lymph node dissection and splenectomy.

**Figure 7:**
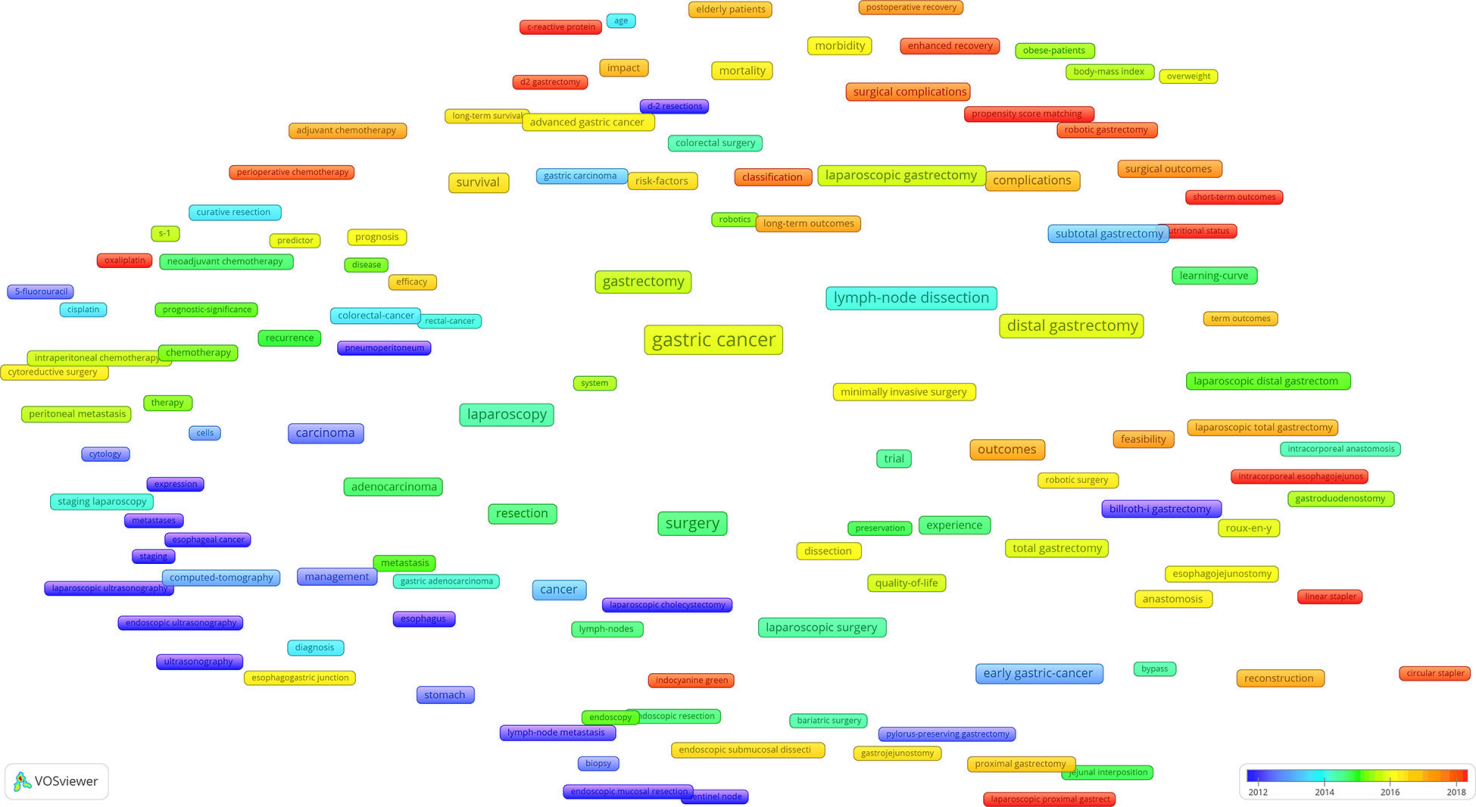
The co-occurrence overlay map based on VOSviewer has different colors according to the average publication years.

In the co-occurrence overlay map in **Figure 7**, the color bar is visible at the lower right corner, and different average publication years correspond to different colors. For example, keywords such as “pneumoperitoneum,” “Billroth I gastrectomy,” and “lymph node metastasis” are blue-purple, mainly appearing near 2012. Meanwhile, keywords such as “laparoscopic proximal gastrectomy,” “esophageal-gastric junction,” and “surgical outcomes” are orange, mainly appearing near 2018, indicating that these fields have become increasingly popular in recent years and may become hotspots in the future.

## 4 Discussion

### 4.1 Publishing trends

In this study, we identified literature describing research on laparoscopic surgery for GC in the last 20 years in the Web of Science. Bibliometric analysis was used to analyze the valuable information regarding the countries and institutions of origin, as well as author keywords in the literature, so as to show the process of development and trends in this research field more intuitively and systematically, and to explore the emerging research hotspots.

In the last 20 years, the number of publications on laparoscopic surgery for GC treatment has increased year by year; especially in the last 5 years, the number of publications has reached new heights. The number of publications after 2017 was 1300, accounting for 46.49% of the total publications. There are two main reasons for this explosive growth: (1) the remarkable curative effect of laparoscopic surgery when used to treat GC; and (2) the increasing popularity of laparoscopic surgery for GC in medical institutions. We will elaborate on the first point later. Regarding the second point, studies have shown that the use of laparoscopic surgery in GC resection has increased over time, especially in hospitals with a large volume of patients (7).

In terms of the distribution of the countries from which the literature was derived, Japan ranked first globally, followed by China and then South Korea. Interestingly, in terms of the total number of citations, these three Asian countries still occupy the top 3 positions (**Table 2**), and the top 10 research institutions and scholars are all from Asian countries (**Tables 3** and **4**). This highlights Asia’s outstanding contribution to and major influence in this field. Notably, Chinese research institutions and researchers have the most recent average publishing years, indicating that increasing attention is being paid to research in this field in China and that such research in this country has particular potential. Indeed, China has developed rapidly in this field. In addition to its high incidence of GC and large disease population, the establishment of the Chinese Laparoscopic Gastrointestinal Surgery Research Group (CLASS) is of great significance. Although this group was established later than the Japan Clinical Oncology Group (JCOG) and the Korean Laparoscopic Gastroenterology Surgery Research Group (KLASS), many of its research results have been widely recognized by the academic community, and there are high expectations regarding its future research. We also found that there is a significant gap between Western countries and Asian countries in the research field of laparoscopic surgery for GC. This may be due to the high incidence rate of GC and the high detection rate of EGC in Asian countries, which enable Asian surgeons to rapidly develop and spread minimally invasive technologies. In contrast, the rate of detection of EGC in Western countries is low, the uses of minimally invasive surgery and endoscopy are less common, the proportion of patients at a locally advanced stage is higher, and the feasibility of laparoscopic resection is low (8, 9). In recent years, researchers in Western countries have also been seeking collaboration with and advice from influential Asian researchers in this field (10, 11).

As revealed by our additional co-authorship analysis, researchers at National Cancer Centre Korea, Shanghai Jiao Tong University, and Shizuoka Cancer Center collaborated most with high-yield institutions (**Figure 4**), suggesting that these three institutions may be centers of collaboration with research institutions globally. At the same time, approximately 98% of high-yield authors are included within the co-authorship network (**Figure 5**), which indicates that high-yield authors are particularly prone to collaborating and that research is relatively concentrated in particular institutions.

### 4.2 Research hotspots

The analysis of high-frequency keywords showed that there are five main hot modules in the research field of laparoscopic surgery for GC: surgical methods for gastric cancer, short-term and long-term efficacy of laparoscopic surgery, guiding role of laparoscopy in the treatment of advanced GC, diagnosis and treatment of EGC, and lymph node dissection.

In terms of surgical methods, since Kitano S. first proposed LADG for EGC in 1994 (4), laparoscopic technology has been widely used in the surgical treatment of GC due to its advantages and safety. These procedures include laparoscopic distal gastrectomy (LDG), laparoscopic total gastrectomy (LTG), and laparoscopic proximal gastrectomy (LPG). Suitable surgical methods can be selected according to the location of the lesion. Robot-assisted gastrectomy (RAG) was first announced in 2003 (12). With the development of robotic equipment and the accumulation of surgical experience, this technology has become increasingly mature. A recent meta-analysis (13) found that RAG was associated with less bleeding and fewer complications, and dissection of more lymph nodes, but longer operating time, higher cost, and no significant difference in long-term outcomes. Although the safety and feasibility of RAG have been verified, there have been few studies on its long-term outcomes, and those that have been performed are of low quality because of the expensive equipment, long learning curve, and availability in only a small number of high-volume centers. Whether the patients can benefit in terms of the survival rate and other aspects still needs to be evaluated by high-quality randomized controlled trials. In the earlier period soon after the introduction of laparoscopic, owing to technical factors and unfamiliarity with in intra-corporeal anastomosis and reconstruction, most surgeons chose to make a small incision in the abdomen for in extra-corporeal anastomosis and reconstruction. With the increase of surgeons’ experience and the improvement of laparoscopic surgical instruments, more minimally invasive procedures such as totally laparoscopic distal gastrectomy (TLDG) began to be widely used, and it has been confirmed that it is safe and feasible to reconstruct the digestive tract in intra-corporeal, such as *via* Billroth-I (14), Billroth-II (15), and Roux-en-Y procedures (16). At the same time, to reduce postoperative complications and improve the quality of life of patients after surgery, research on new gastrointestinal anastomosis and reconstruction has gradually emerged. Delta-shaped anastomosis (17), uncut Roux-en-Y (18), and linear-shaped gastroduodenostomy (LSGD) (19) are not only safe and feasible, but also effectively reduce the incidence of postoperative complications such as reflux esophagitis and alkaline reflux gastritis, thus improving the quality of life of patients.

In terms of short-term and long-term efficacy, the efficacy and safety of LDG and LTG compared with those of traditional laparotomy have been widely recognized. First, the short-term efficacy of LDG on EGC and locally advanced GC is significantly better than that of traditional laparotomy, mainly in terms of shorter hospital stay, less blood loss, and fewer complications, among others (20–22). In terms of long-term efficacy, LDG has a 5-year survival rate similar to that of traditional laparotomy, and is a safe and effective alternative, provided that it is performed by experienced surgeons (23). In a retrospective study, the short-term efficacy of LTG was also better than that of open surgery. Despite the longer operation time, it is associated with a shorter hospital stay, less blood loss, and a lower complication rate (24). In terms of the long-term efficacy, the safety of LTG is comparable to that of open surgery, there is no significant difference in morbidity and mortality, but it is limited to only the treatment of EGC (25, 26). The treatment effect of locally advanced GC remained unclear. A meta-analysis showed that TLDG exhibited advantages in short-term outcomes over LDG, including in terms of blood loss, length of hospital stay, and frequency of analgesic use (27). However, most previous results were from retrospective studies, which are associated with certain limitations. Therefore, prospective studies are needed for further confirmation, and we also look forward to the results of KLASS07, a multicenter prospective trial in Korea. The above research results suggest that laparoscopic surgery for GC has grown rapidly in popularity, which is linked to its remarkable efficacy. Multicenter, prospective randomized controlled studies are being carried out at more and more large medical institutions to provide more evidence-based medical evidence for the use of laparoscopic surgery in the treatment of GC.

In terms of the guiding role of laparoscopy in the treatment of advanced GC, several international guidelines recommend staging laparoscopy for patients with locally advanced GC: CSCO recommends cIII stage patients (28); ESMO recommends IB-III stage patients (29); NCCN recommends T3 and/or N+ patients (30), while Dutch national guidelines recommend patients with ≥ cT3 (31). Staging laparoscopy can make the preoperative staging of patients with advanced GC more accurate, improve the detection rate of peritoneal metastasis, find locally advanced lesions, and detect positivity for exfoliative cells after peritoneal lavage and other nonresectable factors, enabling the selection of appropriate treatment strategies and avoiding unnecessary surgery or ineffective adjuvant treatment (32, 33). At staging laparoscopy, in patients with peritoneal metastasis and/or positivity for exfoliated cells, multiple laparoscopic interventions can be used to reduce clinical symptoms or perform intensive therapy, such as gastrojejunostomy, placement of metal stents, and retention of peritoneal drainage tubes for hyperthermic intraperitoneal chemotherapy (34). With the revision of the guidelines, the use of staging laparoscopy in locally advanced GC has increased significantly (31, 35). A Dutch research team took the lead in conducting a multicenter prospective observational cohort study on the value of staging laparoscopy in patients with locally advanced GC. Its results were consistent with previous findings, and it is believed that staging laparoscopy has significant benefits in staging locally advanced GC (36).

In terms of EGC diagnosis and treatment, endoscopic diagnosis and EGC treatment are the two core hotspots. EGC is an invasive carcinoma located in the mucosa or submucosa, independent of lymph node metastasis. It is usually considered as a stage I tumor in the TNM staging system, including IA and IB (9). With the progress of technology, an increasing number of advanced endoscopic techniques are being applied to the screening of EGC. Compared with traditional white-light endoscopy, chromoendoscopy, magnifying endoscopy with narrow-band imaging (ME-NBI), blue laser imaging, and other advanced endoscopic technologies have significantly improved the diagnostic sensitivity; when considering the examination time, cost, and other factors, ME-NBI appears to be more favored by endoscopists (37). With the development of these new endoscopic diagnostic modalities, some new classification criteria are also gradually being applied in the diagnostic evaluation of EGC to improve diagnostic accuracy. The use of the Parisian classification in white light endoscopy is still an effective screening method, with high accuracy in the diagnosis of EGC. However, some superficial EGC cases with subtle morphological changes are difficult to detect by white light endoscopy. Upon the application of ME-NBI, classification methods such as the Vascular Surface (VS) Classification and Magnifying Endoscopy Simple Diagnostic Algorithm for Early Gastric Cancer (MESDA-G) can be used to supplement the observation of subtle lesions, and the combination with white light endoscopy can improve the diagnostic performance (38, 39). The hotspots of EGC treatment are optimizing the indications of endoscopic resection and evaluating the risk of lymph node metastasis, so as to determine the best treatment plan for EGC patients. Some researchers believe that it is necessary to consider tumor histology in addition to submucosal invasion depth, lymphatic vessel invasion, lesion size, and other factors when deciding to perform endoscopic resection. Histologically differentiated/undifferentiated mixed EGC is associated with low-risk lymph node metastasis(LNM) and good prognosis, and should not be regarded as a factor precluding endoscopic treatment. Meanwhile, the presence of the primitive phenotype in differentiated EGC is a high-risk factor for LNM, which should be evaluated with stricter criteria before endoscopic resection, or additional surgery should be considered after endoscopic resection (40–42). In patients with EGC not eligible for endoscopic resection, laparoscopic surgery can be used as a routine treatment option because of its safety and feasibility. The corresponding findings have been mentioned above.

In terms of lymph node dissection, research on laparoscopic D2 lymph node dissection is a current hotspot. R0 resection combined with D2 lymphadenectomy has been widely accepted as the standard surgical treatment for locally advanced GC in Asian countries (43). Although there are differences in the accepted standard between the East and the West in this regard, with the development of research they have tended to become increasingly similar. In Western countries, surgeons can also complete D2 lymph node dissection with the spleen and pancreas preserved through appropriate training, so it is also recommended for patients with advanced GC in the guidelines of some countries (44). With the standardization of the D2 lymph node dissection process, the direction of research has gradually shifted to quality control and additional area dissection. In some studies, it has been asserted that the quantity and quality of D2 lymph node resection are related to the survival rate, and the main factors associated with a poor prognosis are insufficient resection quantity and a non-standard resection range (45, 46). In this regard, a unique surgical standardization and quality control system has been developed in South Korea through a multicenter, prospective clinical trial (KLASS-02-QC). This system includes the evaluation of unedited surgical video, through which surgeons can improve the level of surgery and quality of lymph node dissection (47). With the progress of technology, Chinese researchers have used indocyanine green (ICG) as an intraoperative guide, which can significantly increase the number of dissected lymph nodes and reduce lymph node non-compliance without increasing surgical complications and operating time. These effects are more pronounced in patients with total gastrectomy (48). In subsequent studies, the team found that the effect of subserosal injection of ICG was similar to that of submucosal injection. Meanwhile, subserosal injection had higher patient satisfaction and lower cost, suggesting that this injection method was a better option (49). In the study of additional range dissection on the basis of D2 lymph node resection, a meta-analysis from China led to the conclusion that D2 lymph node dissection plus complete mesogastrium excision (CME) is safer and more effective than D2 lymph node dissection alone, which was reflected in the removal of more lymph nodes, fewer postoperative complications, a lower local recurrence rate, and an improved 3-year overall survival rate (50). Although D2 lymph node dissection plus abdominal aortic lymph node dissection was safe and feasible, it showed no survival benefit and is associated with a longer operating time and more blood loss (51, 52).

### 4.3 Research trends

According to the co-occurrence overlay map that we established, keywords such as “laparoscopic proximal gastrectomy,” “linear stapler,” “surgical outcomes,” and “esophagogastric junction” have emerged recently, indicating that these fields are increasingly attracting attention. Combined with the current research hotspots, we infer that research on the surgical methods and surgical outcomes of adenocarcinoma of esophagogastric junction (AEG) may become a future research trend.

The incidence of AEG is increasing year by year. In contrast to the case for Siewert type I and type III, there has been no consensus on the surgical method for Siewert type II patients, which is also the classification of most concern to gastrointestinal surgeons. With the progress of research and medical technology, laparoscopic proximal gastrectomy has gradually become the main surgical method for AEG Siewert type II patients, given its advantages of higher survival benefit, and better postoperative quality of life and nutritional status, surgeons prefer to use the double-tract reconstruction method for AEG Siewert type II patients (53–55). However, most of the studies on surgical methods or reconstruction methods have been small-sample retrospective studies, with a lack of large-scale randomized controlled trials to provide higher-level evidence. This also increases our anticipation of the research results of CLASS-10 in China and KLASS-05 in South Korea. There is also controversy over the surgical approach. In a randomized clinical trial with a follow-up period of 10 years, Japanese researchers found that there was no significant difference in 10-year overall survival rate between the transthoracic approach and the transabdominal approach, but the transthoracic approach is not recommended because of its high morbidity and mortality (56). However, a recent meta-analysis suggested that the transthoracic approach and the transabdominal approach are both suitable for Siewert type II patients, and neither of them has significant advantages in terms of short-term and long-term outcomes (57). A multicenter, prospective clinical trial (CARDIA trial) involving many countries may provide new evidence aiding the choice between these two surgical approaches in the future (58).

For AEG, especially Siewert type II patients, owing to the special tumor location, intensive debates are still ongoing about the surgical methods, reconstruction methods, and lymph node dissection range. In recent years, with the maturity of laparoscopic technology and the accumulated experience of surgeons, an increasing number of studies have been conducted, but there is still a lack of consensus about these issues. Our research suggests that laparoscopic surgery for AEG may become an important research topic.

## 5 Conclusion

This study used bibliometric analysis to determine the current research hotspots and research trends in the field of laparoscopic surgery for GC. At present, there are five main topics of research on laparoscopic surgery for GC: surgical methods for GC, short-term and long-term efficacy of laparoscopic surgery, guiding role of laparoscopy in the treatment of advanced GC, diagnosis and treatment of EGC, and lymph node dissection. Along with our findings on the current research hotspots and emerging keywords, we believe that laparoscopic surgery for AEG may become an important research topic in the future.

## 6 Limitations

This study only included core research in the Web of Science, with the literature search being limited to articles written in English. It may thus have ignored other high-quality articles that are not included in this database or were written in other languages.

## Data availability statement

The original contributions presented in the study are included in the article/**Supplementary Materials**. Further inquiries can be directed to the corresponding authors.

## Author contributions

WH was responsible for the conceptualization of the study and the design of the idea. HH and WZW were responsible the writing of the first draft. WZQ was responsible for collecting data and visualizing the results. LG and FY validated the visualization results. ZX was responsible for the editing and standardization of the tables and figures. All authors reviewed the manuscript for important intellectual content and approved the final version for publication.
