# Supplementary material for: Global scientific trends in laparoscopy and gastric cancer in the 21st century: A bibliometric and visual mapping analysis
Source: Front Oncol. 2023 Feb 23;13:1136834. doi: 10.3389/fonc.2023.1136834 (PMC9995981; doi:10.3389/fonc.2023.1136834)
Supplement: Supplementary file 1 [file Table_1.docx]

SUPPLEMENT TABLE Top 30 high-frequency keywords in each country

| **Rank** | **JAPAN** |  | **CHINA** |  | **SOUTH KOREA** |  | **USA** |  |
| --- | --- | --- | --- | --- | --- | --- | --- | --- |
|  | keywords | counts | keywords | counts | keywords | counts | keywords | counts |
| 1 | gastric cancer | 520 | gastric cancer | 459 | gastric cancer | 316 | gastric cancer | 121 |
| 2 | distal gastrectomy | 273 | distal gastrectomy | 281 | gastrectomy | 165 | surgery | 60 |
| 3 | surgery | 263 | surgery | 229 | distal gastrectomy | 165 | distal gastrectomy | 45 |
| 4 | lymph-node dissection | 242 | lymph-node dissection | 202 | laparoscopy | 163 | gastrectomy | 43 |
| 5 | gastrectomy | 168 | gastrectomy | 156 | surgery | 162 | cancer | 38 |
| 6 | laparoscopic gastrectomy | 167 | laparoscopy | 149 | lymph-node dissection | 149 | laparoscopy | 38 |
| 7 | early gastric-cancer | 154 | laparoscopic gastrectomy | 118 | outcomes | 96 | survival | 38 |
| 8 | laparoscopic surgery | 124 | survival | 110 | early gastric-cancer | 92 | carcinoma | 37 |
| 9 | carcinoma | 121 | outcomes | 109 | laparoscopic gastrectomy | 78 | lymph-node dissection | 37 |
| 10 | outcomes | 113 | complications | 106 | complications | 72 | adenocarcinoma | 36 |
| 11 | complications | 98 | total gastrectomy | 83 | mortality | 55 | laparoscopic gastrectomy | 28 |
| 12 | laparoscopy | 95 | resection | 77 | cancer | 55 | outcomes | 26 |
| 13 | billroth-i gastrectomy | 89 | carcinoma | 72 | carcinoma | 51 | resection | 26 |
| 14 | surgical complications | 86 | mortality | 72 | subtotal gastrectomy | 50 | management | 25 |
| 15 | trial | 76 | morbidity | 70 | morbidity | 49 | minimally invasive surgery | 22 |
| 16 | cancer | 74 | advanced gastric cancer | 67 | resection | 48 | staging laparoscopy | 20 |
| 17 | laparoscopic distal gastrectomy | 74 | risk-factors | 61 | learning-curve | 44 | subtotal gastrectomy | 19 |
| 18 | resection | 69 | surgical complications | 58 | survival | 42 | chemotherapy | 16 |
| 19 | classification | 64 | experience | 56 | laparoscopic surgery | 42 | laparoscopic surgery | 14 |
| 20 | feasibility | 62 | surgical outcomes | 56 | trial | 41 | learning-curve | 14 |
| 21 | morbidity | 62 | cancer | 54 | risk-factors | 41 | colorectal-cancer | 13 |
| 22 | survival | 60 | anastomosis | 53 | quality-of-life | 41 | computed-tomography | 13 |
| 23 | dissection | 59 | early gastric-cancer | 52 | experience | 41 | stomach | 13 |
| 24 | mortality | 58 | laparoscopic surgery | 52 | total gastrectomy | 39 | trial | 13 |
| 25 | quality-of-life | 56 | dissection | 51 | anastomosis | 35 | complications | 12 |
| 26 | roux-en-y | 55 | classification | 49 | billroth-i gastrectomy | 33 | morbidity | 12 |
| 27 | anastomosis | 53 | enhanced recovery | 48 | surgical outcomes | 32 | early gastric-cancer | 11 |
| 28 | reconstruction | 50 | trial | 46 | stomach | 30 | experience | 11 |
| 29 | total gastrectomy | 50 | impact | 44 | minimally invasive surgery | 29 | impact | 11 |
| 30 | experience | 48 | subtotal gastrectomy | 43 | recurrence | 28 | peritoneal metastasis | 11 |
